# Effects of Reducing Sedentary Behaviour on Cardiac Structure and Function at Rest and During Exercise: A 6-Month Randomized Controlled Trial

**DOI:** 10.1016/j.cjco.2025.09.005

**Published:** 2025-09-16

**Authors:** Jooa Norha, Maria Saarenhovi, Petri Kallio, Tanja Sjöros, Taru Garthwaite, Saara Laine, Noora Houttu, Kirsi Laitinen, Henri Vähä-Ypyä, Harri Sievänen, Eliisa Löyttyniemi, Tommi Vasankari, Juhani Knuuti, Kari K. Kalliokoski, Ilkka H.A. Heinonen

**Affiliations:** a Turku PET Centre, University of Turku and Turku University Hospital, Turku, Finland; b Department of Clinical Physiology and Nuclear Medicine, University of Turku and Turku University Hospital, Turku, Finland; c Integrative Physiology and Pharmacology Unit, Institute of Biomedicine, University of Turku, Turku, Finland; d Nutrition and Food Research Centre, University of Turku, Turku, Finland; e The UKK Institute for Health Promotion Research, Tampere, Finland; f Department of Biostatistics, University of Turku and Turku University Hospital, Turku, Finland; g Faculty of Medicine and Health Technology, Tampere University, Tampere, Finland

**Keywords:** Sedentary behaviour, cardiac structure, echocardiography, accelerometry, physical inactivity, obesity

## Abstract

**Background:**

Interventional studies on sedentary behaviour (SB) and cardiac health are missing. Therefore, this study investigates the effects of reducing SB on cardiac structure and function in inactive and sedentary adults with metabolic syndrome.

**Methods:**

In this randomized controlled trial, the intervention group (n = 33) aimed at reducing SB by 1 h/d for 6 months. The control group (n = 31) continued their SB and physical activity (PA) as usual. All participants wore accelerometers throughout the study. Echocardiography was performed at rest and during incremental exercise tests before and after the intervention.

**Results:**

No intervention effects were observed in any echocardiographic variables between the randomized groups. However, when participants were regrouped into a less sedentary (mean SB reduction 60 min/d) or a continuously sedentary group, based on their actual measured behaviour change, left ventricular (LV) mass index and end-diastolic diameter decreased more in the less sedentary than in the continuously sedentary group (group x time *P* = 0.045 and 0.020, respectively). Moreover, LV global longitudinal strain during exercise improved in the less sedentary group compared to the continuously sedentary group. Among all participants, the change in light PA was correlated inversely with the change in LV mass index (*r* = –0.32, *P* = 0.026), and the change in standing time was correlated with the change in the early diastolic flow velocity / lateral mitral annular velocity (E/e’) ratio (*r* = 0.28, *P* = 0.048).

**Conclusions:**

A 6-month intervention aimed at reducing SB did not affect cardiac structure or function. However, in participants with successful SB reduction and increased light PA regardless of original randomization, LV mass index may have decreased, and LV function during exercise may have improved.

**Clinical Trial Registration:**

NCT03101228.

## Résumé

**Contexte:**

Il existe un manque d'études interventionnelles sur le comportement sédentaire (CS) et la santé cardiaque. Par conséquent, cette étude examine les effets de la réduction du CS sur la structure et la fonction cardiaques chez des adultes inactifs et sédentaires atteints d'un syndrome métabolique.

**Méthodologie:**

Dans cet essai randomisé contrôlé, le groupe d'intervention (n = 33) visait à réduire le CS de 1h/jour pendant six mois. Le groupe témoin (n = 31) a poursuivi son CS et son activité physique (AP) habituels. Tous les participants ont porté des accéléromètres tout au long de l'étude. Une échocardiographie a été réalisée au repos et pendant un test d'effort progressif avant et après l'intervention.

**Résultats:**

Aucun effet de l'intervention n'a été observé sur les variables échocardiographiques entre les groupes randomisés. Cependant, lorsqu'ils ont été reclassés en un groupe moins sédentaire (réduction moyenne du CS de 60 min/jour) ou en un groupe continuellement sédentaire en fonction de leur changement de comportement réel mesuré, l'indice de masse ventriculaire gauche (VG) et le diamètre télédiastolique ont diminué davantage dans le groupe moins sédentaire que dans le groupe continuellement sédentaire (groupe x temps *p* = 0,045 et 0,020, respectivement). De plus, la déformation longitudinale globale du VG pendant l'exercice s'est améliorée dans le groupe moins sédentaire par rapport au groupe continuellement sédentaire. Parmi tous les participants, le changement dans l'AP légère était inversement corrélé au changement dans l'indice de masse du VG (r = -0,32, *p* = 0,026) et la variation du temps passé debout était corrélé au changement dans le rapport E/e' (r = 0,28, *p* = 0,048).

**Conclusions:**

Une intervention de six mois visant à réduire le CS n'a pas eu d'effet sur la structure ni la fonction cardiaque. Cependant, chez les participants ayant réussi à réduire leur sédentarité et à augmenter leur AP légère, indépendamment de la randomisation initiale, l'indice de masse du VG pourrait avoir diminué et la fonction du VG à l'effort pourrait s'être améliorée.

Enregistrement de l'essai clinique: NCT03101228.

Cardiac dysfunction, or preclinical heart failure, is a prevalent finding among older adults.^1^^,^^2^ Concomitantly, metabolic syndrome, which is a cluster of cardiovascular disease risk factors, such as obesity, hypertension, dyslipidaemia, and dysglycaemia, affects over one-third of adults in the US.^3^ The longitudinal course of developing cardiac dysfunction is closely associated with the presence or development of metabolic syndrome and its components.^1^ Globally, the overall prevalence of heart failure is increasing, and with that increase comes a high economic burden and decreased quality of life.^4^ Therefore, effective prevention strategies are crucial.

Physical activity (PA) is associated with a lower incidence of heart failure, especially in individuals without established atherosclerosis.^5^ Furthermore, observational studies show an increased risk for cardiovascular disease with a high amount of sedentary behaviour (SB).^6^, ^7^, ^8^ Concomitantly, interventions to reduce the level of SB have led to small beneficial changes in cardiometabolic risk factors, such as body composition and glycated hemoglobin concentration.^9^ Additionally, a higher level of SB is associated with an increased risk of hypertension, obesity, and type 2 diabetes,^6^^,^^10^^,^^11^ which are known risk factors for cardiac dysfunction.^2^^,^^12^ Yet, to the best of our knowledge, the evidence on the effects of SB on cardiac structure and function relies exclusively on a few observational studies with inconclusive results.^13^, ^14^, ^15^, ^16^, ^17^ However, these studies suggest that a high amount of SB could be associated with a greater left ventricular (LV) mass.^13^^,^^15^ Thus, randomized controlled trials (RCTs) studying the effects of reducing SB on cardiac structure and function are warranted.

In this study, we investigated whether a 6-month SB reduction intervention affects LV structure, and LV systolic and diastolic function at rest, in 64 physically inactive and sedentary adults with metabolic syndrome. Additionally, LV systolic function was evaluated during an incremental maximal bicycle ergometer test.

## Materials and Methods

The data analyzed in this study consist of secondary outcomes of an RCT conducted at the Turku PET Centre (Turku, Finland) between April 2017 and March 2020. The main outcomes of the study and a more detailed description of the methods are reported elsewhere.^18^^,^^19^ The study protocol was registered at Clinicaltrials.gov (NCT03101228, 05/04/2017), and it was approved by the Ethics Committee of the Hospital District of Southwest Finland (decision number 16/1801/2017). The study was conducted according to the Declaration of Helsinki. Participants gave their informed consent before entering the study.

The study consisted of a 4-week screening phase, during which habitual PA and SB were measured. After the screening, a 6-month intervention period followed. Outcome measures were assessed before and after the intervention.

### Participants

The participants were recruited from the local community. We recruited volunteers who had an elevated risk for cardiovascular disease and who could benefit from a SB-reducing intervention. As reported earlier,^19^ inclusion criteria for the participants were as follows: physical inactivity (< 120 min/wk of self-reported moderate PA); a high amount of sedentary time (≥ 10 h/d or ≥ 60% of daily accelerometer wear time during screening); age 40–65 years; overweight or obesity (body mass index [BMI], 25–40 kg/m^2^); and metabolic syndrome as defined by Alberti et al.^3^ Exclusion criteria were as follows: any diagnosed cardiac disease; uncontrolled hypertension (≥ 160/100 mm Hg); diagnosed diabetes or fasting blood glucose ≥ 7 mmol/L; excessive alcohol consumption; tobacco use; and any condition that could endanger the study procedure.

### Participant characteristics and accelerometry

A total of 151 volunteers underwent a 4-week accelerometer screening period during which their baseline levels of SB, light PA (LPA), moderate-to-vigorous PA (MVPA), and standing were assessed. Hip-worn triaxial accelerometers (UKK AM30, UKK Terveyspalvelut Oy, Tampere, Finland) were used, and the raw acceleration data were analyzed using validated mean amplitude deviation and angle for posture estimation methods.^20^^,^^21^ Using these algorithms, LPA was defined as 1.5 to < 3.0 metabolic equivalents of task (METs; 3.5 mL O_2_/kg/min), and MVPA was defined as ≥ 3.0 METs. During < 1.5-MET activities, body posture was classified as either standing or SB (ie, sitting, lying, or reclining). The accelerometer data were analyzed in 6-second epochs. Non-wear time was identified if the acceleration in all 3 axes remained within the 187.5 milligravity range for ≥ 30 minutes, and a valid day of accelerometry was defined as 10–19 hours of wear time.

Stature and body mass were measured, and BMI was calculated. Body surface area was calculated using the Du Bois formula: (0.007184 x weight^0.425^ x height^0.725^).^22^ Additionally, air displacement plethysmography (Bod Pod, COSMED USA, Concord, CA) was performed to assess body fat percentage and fat-free mass.

### Intervention

The eligible participants were randomized into the intervention and control groups in a 1:1 ratio. A statistician performed the randomization using random permuted block randomization (block size, 44) for men and women separately using SAS (version 9.4 for Windows; SAS Institute, Cary, NC).

All participants wore accelerometers on the hip (Movesense, Suunto, Vantaa, Finland) during the 6-month intervention. The accelerometer data were analyzed using the same algorithms used during screening. The participants could monitor their daily SB and PA on a mobile phone application (ExSed, UKK Terveyspalvelut Oy). In addition, individual goals for SB, LPA, MVPA, and standing were set on the application, based on the measured activity behaviour during the screening period. The aim of the intervention group was to reduce SB by 1 h/d. Correspondingly, 1 h/d was added to LPA, MVPA, and standing, according to personal preferences. However, a maximum of 20 min/d was added to MVPA. The goals for the control group were set equal to the levels in the screening period.

The intervention group received a 1-hour individual counselling session to discuss alternative ways to reduce SB. For example, the use of standing desks, walking during phone calls, or taking the stairs instead of an elevator were encouraged. During the 6-month intervention, the participants were contacted monthly via telephone, and they visited the research centre at the midpoint of the study to ensure that the accelerometers were functioning and to get support in making the behavioural change. Both groups were advised not to take up any new physical exercise training habits during the intervention period.

### Echocardiography

Standard echocardiographic assessments were performed by 2 experienced clinical physiology specialist doctors (M.S. and P.K.). The examiners were blinded to the group allocation. LV end-diastolic diameter and LV wall thickness (posterior, septum) were measured from parasternal long-axis M-mode imaging at end diastole. Relative wall thickness was calculated as LV posterior wall thickness ∗ 2 / LV end-diastolic diameter. LV mass and mass index (indexed to body surface area) were estimated from M-mode imaging using the American Society of Echocardiography formula (0.8 x 1.04 x [{septum thickness + LV end-diastolic diameter + posterior wall thickness}^3^ – LV end-diastolic diameter^3^] + 0.6).^23^ LV end-systolic and end-diastolic volumes and LV ejection fraction were measured from the apical 2- and 4-chamber views using the Simpson’s biplane method. Stroke volume (end-diastolic volume [mL] – end-systolic volume [mL]) and cardiac output (stroke volume x heart rate) were calculated. Left atrial and aortic root diameters were measured from the parasternal long-axis M-mode imaging at end diastole. The left atrial end-systolic volume index was estimated using the biplane area-length method from the apical 2- and 4-chamber views, indexed to body surface area. Apical 4-chamber pulsed wave Doppler imaging was used to measure the peak early diastolic (E) and atrial contraction (A) flow velocities, and the E/A ratio was used as a marker of diastolic function. Lateral early diastolic mitral annulus velocity (e’) was measured from the apical 4-chamber view, and the ratio of E/e' was used as a measure of diastolic function. LV global longitudinal strain (GLS) was measured using the speckle tracking method by averaging the GLS from the apical 2-, 3- and 4-chamber views. Frame rate was set adequate for GLS measurements (> 50 frames per second). The sufficiency of image quality was judged visually by the examiners.

Echocardiography was performed additionally during a maximal incremental exercise test. The test was performed as previously described using a recumbent bicycle ergometer (eBike EL Ergometer with Case v6.7; GE Medical Systems Inc., Milwaukee, WI).^19^ In brief, the test was initiated at 25 W, and the load was increased every 3 minutes by 25 W until either volitional exhaustion or a medical reason for termination (eg, abnormally high blood pressure) occurred. Breath-by-breath respiratory gas measurements (Vyntus CPX, CareFusion, Yorba Linda, CA) and heart rate were used to verify maximal effort.^19^ After 2 minutes at each stage of the exercise test, LV GLS was assessed as described above.

One-lead electrocardiography was used to identify the cardiac cycle and heart rate during the echocardiographic measurements. The echocardiography was performed using Vivid E9 (GE Vingmed Ultrasound AS, Horten, Norway), and the image analysis was performed using the Echopac plugin in ViewPoint version 6.12 (GE Healthcare, Solingen, Germany).

### Statistical analyses

We recruited 64 participants based on an *a priori* power calculation for the main outcome of the study (whole-body insulin sensitivity, reported previously by Sjöros et al.^18^).

Baseline data are presented as mean (standard deviation) if not stated otherwise. Intervention results are presented as model-based means (95% CI). All participants were included in the analyses, regardless of missing data. The intervention analyses were performed using linear mixed models for repeated measurements in SAS (version 9.4, SAS Institute). The model included group (between-subject variable), time (within-subject variable) and group∗time; sex was also included in the model. The same model was used for both resting and exercise outcomes. The normal distribution of the Studentized residuals was inspected visually. Unstructured or compound symmetry covariance structure was selected based on the Akaike information criterion. Pairwise comparisons were adjusted using the Tukey-Kramer method. A 2-tailed *P*-value of < 0.05 was considered statistically significant.

As an additional analysis, we re-divided the participants based on the measured change in SB into a less sedentary (SB decrease of ≥ 3%-points of accelerometer wear time, or about 27 min/d with 15 hours of wear time) and a continuously sedentary (SB decrease of < 3%-points or an increase in SB) group. The participants with missing accelerometer data (n = 8) were allocated according to their original randomization. The cutoff of 3%-points was chosen, as it resulted in relatively equally-sized groups (n = 34 and 30, respectively), as in our previous work.^18^^,^^19^

Finally, we calculated the Pearson correlations between the changes in the accelerometer variables, body composition, and cardiac variables among all participants. The correlation analyses were conducted in IBM SPSS Statistics (version 28.0, IBM, Armonk, NY).

To assess the relationship between body composition and exercise GLS image quality, we compared the differences in BMI between the participants who had vs those who did not have sufficient-quality exercise GLS data using 1-way analysis of variance in JMP (Student Edition, version 18.2.1, SAS Institute).

## Results

**Figure 1:**
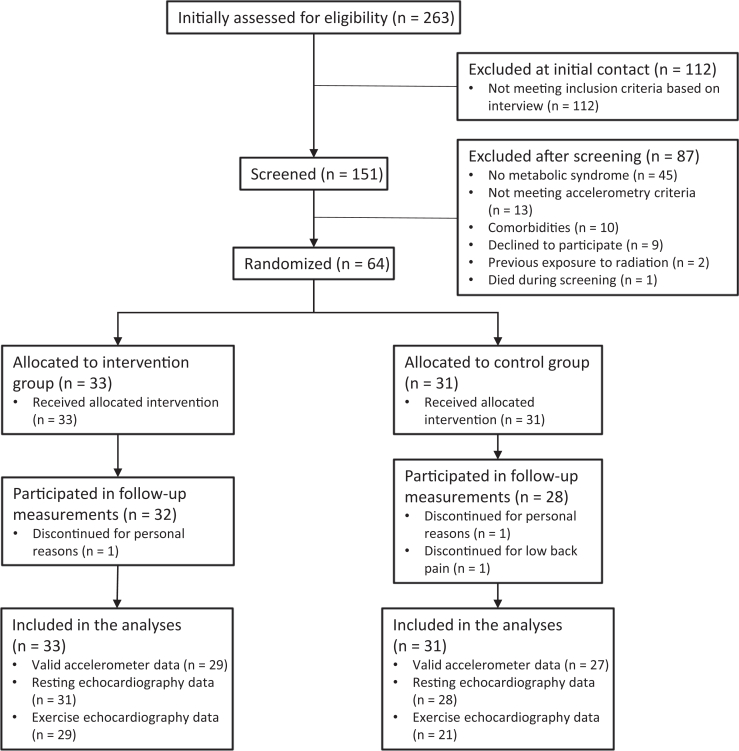
Consolidated standards of reporting trials (CONSORT) flow chart.

A total of 263 volunteers were initially interviewed via telephone or e-mail, of which 151 took part in the screening measurements. The 64 participants deemed eligible based on the screening were then randomized into the intervention (n = 33) and control (n = 31) groups. In the intervention group, one participant discontinued due to personal reasons, and one was excluded from the echocardiographic analyses due to atrial fibrillation. Three participants in the control group discontinued due to personal reasons or low back pain (Fig. 1). The participant characteristics are described in Table 1. Baseline echocardiographic variables are described in Table 2.

**Table 1:** Participant characteristics

Characteristic	Intervention (n = 33)	Control (n = 31)
Females, n (%)	20 (60.6)	17 (54.8)
Age, y	59.3 (6.01)	57.2 (7.5)
Body mass, kg	92.4 (16.6)	94.1 (15.8)
BMI, kg/m^2^	31.5 (4.0)	31.7 (4.6)
Waist circumference, cm	111.1 (11.6)	110.7 (11.1)
Body fat, %	43.1 (8.0)	43.1 (8.0)
Fat mass, kg	39.8 (10.4)	40.9 (11.1)
FFM, kg	52.6 (11.9)	53.2 (9.8)
VO_2_max, mL/kg/min	22.65 (5.05)	22.76 (4.33)
Maximal heart rate, beats/min	159 (15)	152 (16)
Accelerometry during screening, days	25.8 (3.7)	25.7 (3.4)
Wear time, h/d	14.47 (0.96)	14.60 (1.00)
Sedentary time, h/d	10.02 (0.92)	10.06 (1.11)
Standing time, h/d	1.81 (0.61)	1.76 (0.57)
LPA, h/d	1.67 (0.40)	1.81 (0.48)
MVPA, h/d	0.96 (0.31)	0.97 (0.34)
Sedentary proportion, %/wear time	69.2 (5.6)	69.8 (6.6)
Standing proportion, %/wear time	12.4 (3.9)	12.1 (3.9)
LPA proportion, %/wear time	11.6 (2.6)	12.4 (3.0)
MVPA proportion, %/wear time	6.7 (2.2)	6.7 (2.3)
Steps/d	5203 (1910)	5091 (1760)
Sedentary breaks/d	28 (8)	29 (8)

Presented as mean (standard deviation), unless otherwise stated. BMI, body mass index; FFM, fat-free mass; LPA, light physical activity; MVPA, moderate-to-vigorous physical activity; VO_2_max, maximal oxygen uptake.

**Table 2:** Baseline echocardiographic values in the total study sample and intervention and control groups

Measure	All	Intervention	n	Control	n
LV posterior wall, mm	8.6 (1.2)	8.6 (1.4)	32	8.6 (1.1)	31
LV septum, mm	8.5 (1.3)	8.4 (1.3)	32	8.6 (1.3)	31
LVEDD, mm	52.4 (4.8)	52.9 (5.5)	32	51.8 (4.1)	31
Relative wall thickness	0.33 (0.05)	0.32 (0.05)	32	0.33 (0.05)	31
LV mass, g	190.5 (56.1)	194.2 (66.9)	32	186.6 (43.0)	31
LV mass index, g/m^2^	79.5 (16.6)	80.9 (19.3)	32	78.0 (13.3)	31
LVEDV, mL	90.2 (22.3)	88.9 (25.8)	32	91.6 (18.3)	31
LVESV, mL	35.0 (13.6)	35.3 (16.5)	32	34.8 (10.1)	31
LV stroke volume, mL	55.2 (13.3)	53.6 (14.6)	32	56.8 (11.8)	31
Cardiac output, mL/min	3912 (914)	3757 (943)	31	4070 (870)	30
LA diameter, mm	39.9 (4.8)	39.6 (4.8)	32	40.2 (4.7)	31
LAESV index, ml/m^2^	26.8 (7.7)	27.4 (8.8)	31	26.2 (6.5)	29
Aortic root, mm	33.6 (3.6)	33.1 (3.3)	32	34.0 (3.9)	31
LVEF, %	63 (4)	63 (4)	32	63 (5)	31
GLS, %	–18.4 (2.6)	–18.0 (2.9)	31	–18.9 (2.2)	28
E, cm/s	0.69 (0.16)	0.67 (0.14)	32	0.70 (0.18)	31
A, cm/s	0.74 (0.16)	0.77 (0.17)	32	0.72 (0.15)	31
E/A ratio	0.96 (0.28)	0.91 (0.26)	32	1.01 (0.29)	31
Lateral E/e’ ratio	7.5 (1.8)	7.6 (1.9)	32	7.4 (1.7)	31

A, atrial contraction flow velocity; E, early diastolic flow velocity; e’, lateral mitral annular velocity (cm/s); EDD, end diastolic diameter; EDV, end diastolic volume; EF, ejection fraction; ESV, end systolic volume; GLS, global longitudinal strain; LA, left atrium; LV, left ventricle.

### Intervention effects

#### Accelerometry and anthropometrics

The detailed PA and SB results of the intervention have been published previously.^18^ In brief, the intervention group reduced their SB by an average of 40 min/d and increased their MVPA by 20 min/d, whereas no changes in the control group were observed (group∗time *P* < 0.01 for both). LPA increased in both groups by 10 min/d (time *P* = 0.001). Both study groups increased their daily step count, but the change was significantly higher in the intervention group (intervention, +3300 vs control, +1600 steps/d; group∗time *P* = 0.001).

As reported previously,^18^ no between-group differences in BMI, body mass, waist circumference, or body fat percentage were observed, although a slight improvement (eg, a decrease in body mass of ∼1 kg) in all of these was observed among all participants.

#### Cardiac structure and function at rest

**Figure 2:**
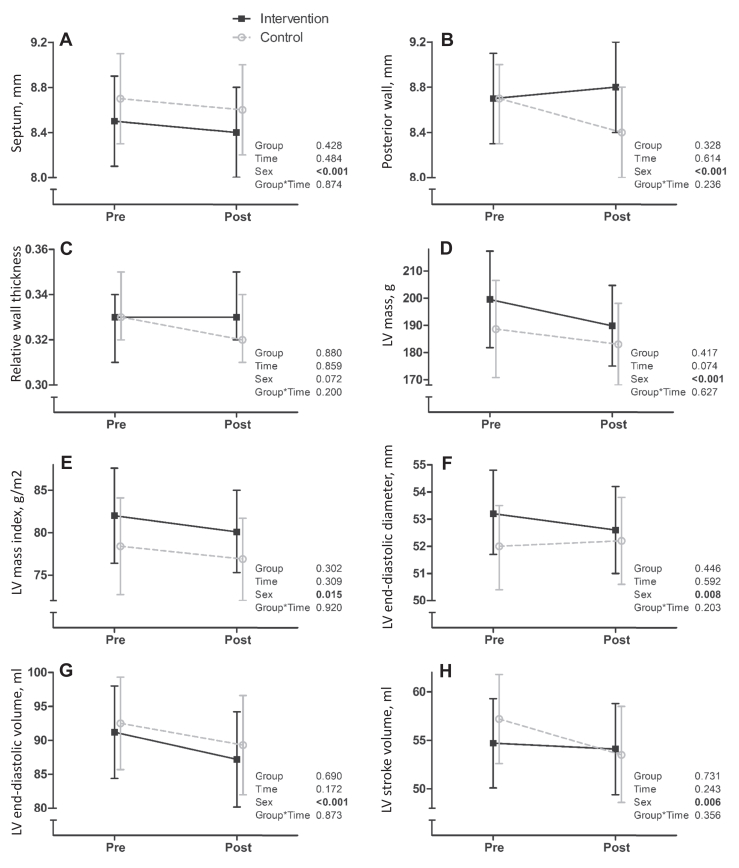
Intervention effects on (**A**) septal thickness (mm), (**B**) posterior wall thickness (mm), (**C**) relative wall thickness, (**D**) left ventricular (LV) mass (m), (**E**) LV mass index (m/g^2^), (**F**) LV end-diastolic diameter (mm), (**G**) LV end-diastolic volume (ml), and (**H**) LV stroke volume (mL). **Black solid lines** represent the intervention group, and **grey dotted lines** represent the control group.

**Figure 3:**
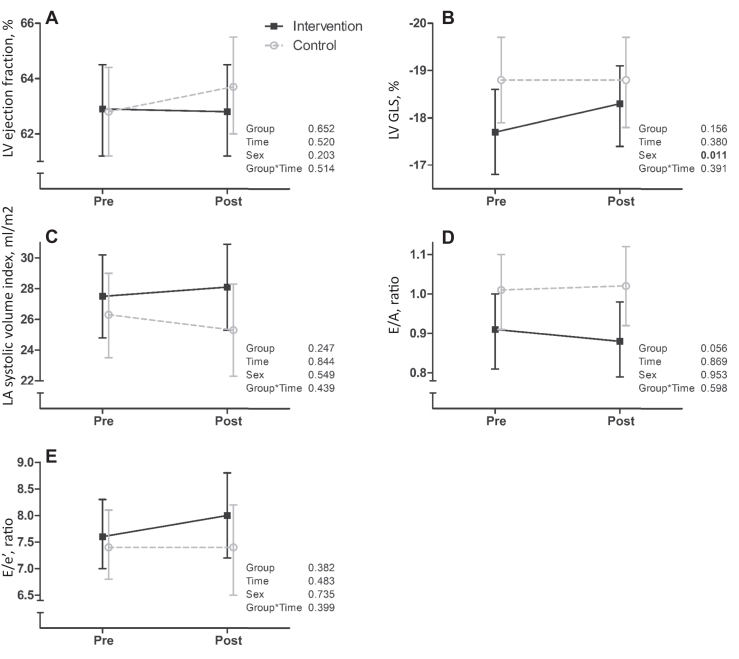
Intervention effects on (**A**) left ventricular (LV) ejection fraction (%), (**B**) LV global longitudinal strain (GLS; %), (**C**) left atrial (LA) systolic volume index (mL/m^2^), (**D**) early diastolic flow velocity / atrial contraction flow velocity (E/A) ratio, and (**E**) E/ lateral mitral annular velocity (E/e’) ratio. **Black solid lines** represent the intervention group, and **grey dotted lines** represent the control group.

No statistically significant changes were observed in cardiac wall thicknesses, diameters, masses, or volumes. Similarly, no statistically significant changes were observed in LV diastolic function markers (E, A, E/A, or E/e’). Finally, no statistically significant changes were observed in LV systolic function markers (ejection fraction, GLS, stroke volume, or cardiac output). The resting echocardiographic measurements in the intervention and control groups before and after the intervention are presented in Figures 2 and 3, and the corresponding numerical estimates are presented in Supplemental Table S1.

#### LV GLS during exercise

The number of sufficient-quality data points on LV GLS decreased when the workload increased (Table 3), and the participants who did not have sufficient-quality LV GLS data had a higher BMI compared to the participants with sufficient-quality data (Supplemental Table S2). We did not observe any statistically significant changes in the GLS measured during exercise. The exercise GLS estimates are presented in Table 3.

**Table 3:** Effects of reducing sedentary behaviour on global longitudinal strain during exercise testing according to the original group allocation and in the additional analysis groups

Allocation	GLS at 25 W			GLS at 50 W			GLS at 75 W			GLS at 100 W			GLS at 125 W		
	Pre	Post	Group∗ Time *P*	Pre	Post	Group∗ Time *P*	Pre	Post	Group∗ Time *P*	Pre	Post	Group∗ Time *P*	Pre	Post	Group∗ Time *P*
**Original group allocation**															
Intervention	–17.8 (–16.6, –19.1)	–19.5 (–18.2, –20.8)	0.131	–17.7 (–16.4, –19.0)	–19.1 (–17.7, –20.6)	0.277	–19.0 (–17.7, –20.2)	–19.3 (–18.0, –20.5)	0.993	–17.8 (–16.3, –19.4)	–18.7 (–17.2, –20.2)	0.470	–15.7 (–13.9, –17.4)	–17.8 (–16.0, –19.5)	0.172
n	25	21		26	21		22	21		15	18		15	15	
Control	–18.8 (–17.4, –20.1)	–18.8 (–17.3, –20.2)		–18.9 (–17.2, –20.5)	–19.0 (–17.4, –20.6)		–18.5 (–17.1, –19.9)	–18.8 (–17.4, –20.2)		–18.6 (–17.1, –20.0)	–18.7 (–17.1, –20.3)		–17.8 (–15.7, –20.0)	–18.0 (–15.6, –20.4)	
n	19	17		16	16		16	17		17	14		10	7	
**Additional analysis**															
Less sedentary	–17.6 (–16.4, –18.8)	–19.5 (–18.2, –20.8)	**0.032**	–17.5 (–16.2, –18.9)	–19.1 (–17.7, –20.5)	0.151	–18.4 (–17.3, –19.6)	–19.0 (–17.8, –20.3)	0.419	–17.1 (–15.7, –18.5)	–18.8 (–17.4, –20.3)	**0.015**	–15.0 (–13.5, –16.6)	–17.7 (–16.0, –19.4)	**0.029**∗
n	25	21		25	21		24	21		17	16		16	12	
Continuously sedentary	–19.1 (–17.7, –20.4)	–18.7 (–17.3, –20.1)		–19.0 (–17.5, –20.6)	–19.0 (–17.4, –20.6)		–19.4 (–17.9, –20.9)	–19.1 (–17.7, –20.5)		–19.4 (–17.9, –20.9)	–18.8 (–17.3, –20.2)		–19.1 (–17.1, –21.2)	–19.0 (–17.0, –20.9)	
n	19	17		17	16		14	17		15	16		9	10	

Sex was significant (*P* < 0.05) in all models except for the GLS at 25 W and 125 W with the original group allocation, and at 25 W, 100 W, and 125 W in the additional analysis. GLS, global longitudinal strain of the left ventricle.

∗ Group *P* = 0.026, time *P* = 0.050.

### Additional analyses

When the participants were divided according to the actual measured change in SB (≥ 3%-point decrease vs < 3%-point decrease or increase in SB), the less sedentary group reduced their SB by 60 min/d and increased their standing, LPA, and MVPA by about 20 min/d each.^24^ SB, LPA, and MVPA did not change statistically significantly in the continuously sedentary group, but standing decreased by 18 min/d.^24^

**Figure 4:**
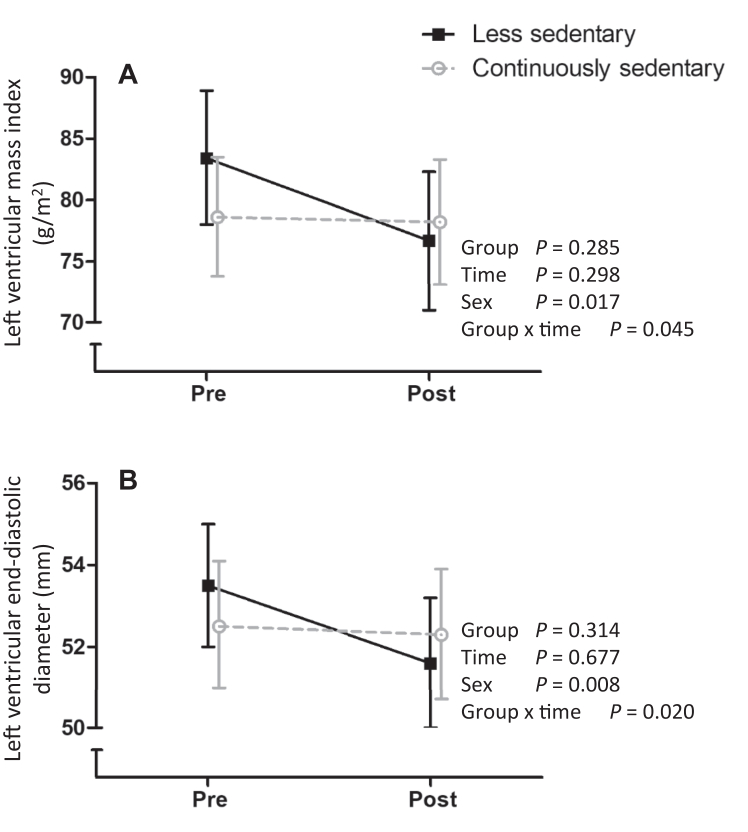
Results from the additional analyses on (**A**) left ventricular mass index (g/m^2^) and (**B**) left ventricular end-diastolic diameter (mm). **Black solid lines** represent the participants who successfully reduced sedentary behaviour by ≥ 3%-points, and **grey dotted lines** represent the participants who either increased their sedentary time or decreased it by < 3%-points.

In the secondary SB-reduction-based groups, statistically significant differences in the change in LV diastolic diameter (group∗time *P* = 0.020) and LV mass index (*P* = 0.045) were observed, in favour of the less sedentary group (Fig. 4). In addition, LV mass tended to decrease in the less sedentary group compared to the continuously sedentary group (*P* = 0.078). All of the resting echocardiographic estimates in the additional analyses are presented in Supplemental Table S3.

During exercise, LV GLS at 25 W, 100 W, and 125 W improved (ie, decreased) in the less sedentary group, whereas an opposite trend or no change was observed in the continuously sedentary group (group∗time *P* = 0.015-0.032; Table 3). The residual histograms from the additional analysis models that were statistically significant are presented in Supplemental Figure S1.

The change in LPA was correlated inversely with the change in LV mass index (*r* = –0.32, *P* = 0.026), and the change in GLS at 75 W (*r* = –0.39, *P* = 0.039). Moreover, the changes in LPA and MVPA were correlated with the change in aortic root dimension (*r* = 0.38 and *r* = 0.28, *P* = 0.01 and *P* = 0.05, respectively). Additionally, the change in standing was correlated positively with the change in the E/e’ ratio (*r* = 0.28, *P* = 0.048) and was correlated negatively with the changes in GLS at 100 and 125 W (*r* = –0.56 and r = –0.54, *P* = 0.008 and *P* = 0.048, respectively). Finally, the change in steps was correlated negatively with the changes in GLS at 25 and 50 W (*r* = –0.41 and *r* = –0.46, *P* = 0.024 and *P* = 0.012, respectively). The correlation coefficients are presented in Supplemental Tables S4 and S5.

## Discussion

In this study, we showed that an individually tailored intervention aimed at reducing SB by 1 h/d for 6 months did not alter the cardiac structure or function in physically inactive and sedentary adults with metabolic syndrome. However, when focusing on the participants with vs without successful reduction in SB, we observed that successfully reducing SB (ie, by 60 min/d throughout the 6-month period, on average) may have decreased LV mass index and end-diastolic diameter. In addition, even though LV GLS during exercise did not change in the original groups, it seemed to improve among the participants who successfully reduced SB.

To our best knowledge, this RCT is the first to investigate the effects of SB reduction, without increasing exercise training, on cardiac structure and function. Nevertheless, the association between SB and cardiac structure and function has been reported in a few previous studies with somewhat mixed results.^13^, ^14^, ^15^ The most consistent finding in these studies is the positive association between SB and LV mass (or mass index).^13^^,^^15^ This interventional study supports the previous associative findings and builds upon them by suggesting that successfully reducing SB may decrease LV mass index. Albeit the data are interventional, a crucial point to understand is that the additional analyses in this study were performed regardless of the original group allocation, which abandons the benefits of randomization and limits causal inference. Additionally, we observed a decrease in LV mass index when LPA was increased, paralleling the results of a previous cross-sectional study in which LPA was associated negatively with LV mass index.^15^ Given that LV hypertrophy (ie, increased mass) increases the risk for incident heart failure,^25^ successfully reducing SB might decrease the risk of heart failure. Moreover, a recent observational study of almost 90,000 individuals found that accelerometer-measured SB in the highest quartile was associated with a 45% higher risk for heart failure compared to the risk in individuals with SB in the second quartile; this association remained significant when examining only individuals whose weekly MVPA exceeded the guideline-recommended 150 minutes, as measured by accelerometry.^8^ An important finding is that although higher-intensity PA, especially more vigorous exercise, is generally associated with an enlarged heart, the association seems to be paradoxical in a hypertensive heart—in fact, PA seems to reverse LV hypertrophy.^26^

An interesting point to note is that cross-sectional results supporting the possible effect of SB on LV mass have been reported previously in adolescents.^27^ Moreover, cumulative SB during an 8-year follow-up associated with higher cardiac work in adolescents, indicating higher LV stress and, therefore, an increased risk for LV hypertrophy.^28^ However, previous intervention studies that reported LV hypertrophy reversal with exercise training have used a notably higher dose of PA (eg, 3 times per week, 60%-80% of maximum heart rate) than that in our SB reduction intervention.^29^, ^30^, ^31^ Taken together, these findings indicate that more research on the effects of lower-intensity PA on cardiac structure is needed.

In agreement with the LV mass index decrease following a successful SB reduction, we observed a decrease in LV end-diastolic diameter in the less-sedentary group compared to the continuously sedentary group. However, although the mean LV end-diastolic volume tended to decrease more in the less-sedentary group, the group∗time was not statistically significant (*P* = 0.411). This discordance may be explained by the biplane measurement, which estimates the LV volume using 2 imaging planes, as opposed to the single measurement of LV diameter. Moreover, although not reported, as it is no longer recommended, we observed a decrease in end-diastolic and systolic volumes estimated from parasternal long-axis M-mode imaging (group∗time *P* < 0.05 for both in the additional analysis). Therefore, the finding of decreased LV end-diastolic diameter with successful SB reduction should be assessed cautiously and interpreted as a preliminary and hypothesis-generating finding.

Although the participants in this study had several cardiovascular risk factors, they were free of diagnosed cardiac diseases. Therefore, an interesting finding is that a successful SB reduction improved GLS during several stages of the exercise test in the additional analysis. In fact, GLS during exercise is a sensitive tool to detect subclinical changes in myocardial function that may not be present at rest.^32^ This study is important in that it is the first SB-related study to include cardiac measurements during exercise. Therefore, our findings may be considered hypothesis-generating. However, a previous cross-sectional study on a healthy subsample of Hispanic and/or Latino adults reported that higher SB levels were associated with poorer LV GLS at rest.^15^ Although no exercise measurements were performed in that study, the direction of the association supports our findings on improved LV GLS during exercise when SB was successfully reduced.

Physiologically, the findings of the present study are in line with our previous findings. In the main analyses, we did not find any intervention effects on either blood pressure at rest or during graded exercise, or cardiorespiratory fitness.^19^^,^^33^ However, if the participants were able to increase PA, their blood pressure during low-to-moderate intensity exercise decreased and cardiorespiratory fitness increased.^19^^,^^33^ As a result, the daily blood pressure load (eg, during household tasks) would theoretically decrease, which could induce beneficial LV remodelling.^34^^,^^35^ Similarly, in non-athletic (mean maximum volume of oxygen uptake [VO_2_max], 28.8 mL O_2_/min/kg) prehypertensive individuals, a higher fitness level was associated with lower LV mass index (*r* = –0.44).^35^ Therefore, a plausible possibility is that even non-exercise PA may be sufficient at inducing beneficial cardiac remodelling. Previous observational and interventional studies also show that SB could have detrimental effects on cardiovascular disease risk factors, such as the risk for incident diabetes or body adiposity,^9^^,^^36^ which further supports the possibility for improved cardiac structure with successful SB reduction.

Among all participants, the change in standing during the 6 months was correlated positively with the change in E/e’, indicating an increased LV filling pressure, or a decline in diastolic function, when standing was increased. This finding is in line with our previous findings indicating that increased standing is associated with adverse changes in cardiorespiratory fitness and BMI.^19^^,^^37^ We speculate that if the increased standing comes at the cost of reduced PA, increasing standing can be associated with adverse health effects. However, prolonged standing at work is associated with an adverse diastolic blood pressure profile in the 24-hour ambulatory measurement,^38^ which could explain the adverse association between increased standing and decreased diastolic function. In contrast to this finding, we also observed that increased standing was associated with improved (ie, decreased) GLS during exercise testing. The reason for these seemingly opposite results remains elusive and requires further investigation.

Finally, we observed that among all participants, the change in aortic root diameter was correlated with the change in LPA and MVPA, meaning that when PA increased, the aortic root size tended to increase, too. Although in the context of PA, a larger aortic size generally would be related to elite athleticism,^39^ a previous twin-study suggests that higher PA is associated with a larger upper abdominal and distal aorta diameter even among non-athletic adults.^40^ Therefore, our results among physically inactive adults with metabolic syndrome and overweight/obesity are in line with the previous findings in healthier and more active adults. Rather than being a disadvantageous adaptation, the increase in aortic diameter could be a result of the increased blood flow demand during PA. Nevertheless, as this was only a correlative finding, further studies are needed.

The intervention was aimed at reducing daily SB by 1 hour for 6 months. However, the mean change during the 6-month intervention was –40 min/d, which is less than originally intended but somewhat expected with a relatively long-duration behavioural intervention. Previous studies have reported similar achieved reductions in SB with similar interventions.^41^^,^^42^ Changing activity behaviours long term may be difficult, and possibly, the intervention should be modified during the follow-up to support sustained behaviour change. Moreover, to achieve sustained behaviour change, prolonged interventions are needed. For example, an RCT among 212 patients with coronary artery disease reported that a 12-week intervention for SB reduction was successful in reducing SB, but this effect was lost at the 3-month follow-up.^43^ In addition, no benefits from the 12-week intervention were observed on any cardiovascular risk factors.^43^

When designing future studies that utilize exercise echocardiography, the type of exercise should be taken into consideration. We used a recumbent cycle ergometer instead of an upright ergometer to ensure the best possible echocardiography quality. This activity does increase cardiac preload as venous return from the lower extremities is increased compared to an upright ergometer. However, we did not measure stroke volume during exercise, and GLS is a relative value that most likely was not notably affected by the exercise posture.

Considering that the LV mass index decreased by 4.8 g/m^2^ (95% CI, 4.2, 5.4) among the participants who successfully reduced SB, and the wide range of normal LV mass indices (ie, 49-115 and 43-95 g/m^2^ for males and females, respectively^23^), the observed change among those participants was relatively minor. Similarly, LV GLS during exercise improved approximately 10% in the less sedentary group, which is less than the proposed > 15% change for a clinically important difference in resting GLS.^44^ Unfortunately, to our best knowledge, no minimal clinically important difference for LV mass index has been published, and the > 15% change in resting GLS has been proposed for monitoring the cardiotoxicity of cancer treatments.^44^ However, a reasonable assumption is that if our findings were to be replicated, the magnitude of change would be small, albeit encouraging. This assumption is also in line with the finding of small changes in cardiovascular risk factors with SB reduction.^9^

### Strengths and limitations

The strengths of this study are the 6-month duration and the RCT setting. In addition, the daily accelerometer monitoring of SB and PA throughout the intervention is exceptional. Moreover, the accelerometer data were analyzed in 6-second epochs, to capture practically all meaningful movement behaviours. In addition, the study participants (ie, physically inactive middle-aged adults with metabolic syndrome) represent a population that would likely benefit from interventions (such as SB reduction) designed to improve cardiovascular health. However, as the present analyses were based on secondary outcomes of the whole trial, the statistical power of the present analyses may have been inadequate. Yet, the study presents novel results that should be interpreted as hypothesis generating. Although echocardiography is a valid tool to measure cardiac structure and function, the measurement is sometimes technically difficult in overweight and obese individuals, especially during exercise testing. As a result, we were able to obtain adequate exercise echocardiographic data in only 26 of the 32 study completers in the intervention group, and 19 of the 28 completers in the control group. Moreover, we observed that the participants who had sufficient-quality exercise GLS data were leaner than the participants without sufficient-quality data. This difference may have caused bias in the results. However, our findings highlight the importance as well as the difficulty of performing exercise measurements in addition to the conventional resting measurements. Future studies also should consider including measurements of diastolic function during exercise, as it was not measured in this study. Moreover, although echocardiographic findings can have prognostic value,^25^ data on the effects of reducing SB on incident cardiovascular events or mortality are needed.

Notably, only LV mass index and not LV mass was reduced to a statistically significant degree in the secondary analyses, which raises the question of whether body size (ie, surface area) affected the results. However, BMI and body mass decreased in the less-sedentary group more than in the continuously sedentary group (body mass decreased 1.3 kg [95% CI –2.5, –0.1] and BMI decreased 0.4 kg/m^2^ (–0.9, –0.01) more in the less-sedentary group; group∗time *P* < 0.05) and the changes in body surface area were not different across groups (group∗time *P* = 0.101; data not shown). Moreover, when we replicated the analyses by additionally adjusting for body mass, the results remained mostly similar. Therefore, the observed improvement in LV mass index likely was due to both reduced SB and consequent PA increase, as well as improvement in body composition.

Finally, due to the nature of the behavioural intervention, blinding of the participants was not possible (see Supplemental Appendix S1). Having all participants be aware of their group allocation may have led to unintended health behaviour changes in both groups.

## Conclusions

An intervention aimed at a daily reduction in SB did not improve cardiac structure or function. However, when SB was successfully reduced and daily non-exercise activities were increased, regardless of original randomization, LV mass index may have decreased and LV function during exercise may have improved, which could be beneficial for cardiac health. Further studies to test this hypothesis are warranted.
