# Supplementary material for: Effects of Reducing Sedentary Behaviour on Cardiac Structure and Function at Rest and During Exercise: A 6-Month Randomized Controlled Trial
Source: CJC Open. 2025 Sep 16;8(1):69–81. doi: 10.1016/j.cjco.2025.09.005 (PMC12925755; doi:10.1016/j.cjco.2025.09.005)
Supplement: Supplementary Material [file mmc1.docx]

**Effects of Reducing Sedentary Behaviour on Cardiac Structure and Function at Rest and During Exercise: A Six-month Randomised Controlled Trial**

**Supplementary Material**

Jooa Norha^1^*, Maria Saarenhovi^2^, Petri Kallio^2^, Tanja Sjöros^1^, Taru Garthwaite^1^, Saara Laine^1^, Kirsi Laitinen^3^, Noora Houttu^3^, Henri Vähä-Ypyä^4^, Harri Sievänen^4^, Eliisa Löyttyniemi^5^, Tommi Vasankari^4, 6^, Juhani Knuuti^1^, Kari K. Kalliokoski^1^, Ilkka H. A. Heinonen^1^

1 Turku PET Centre, University of Turku and Turku University Hospital, Turku, Finland
2 Department of Clinical Physiology and Nuclear Medicine, University of Turku and Turku University Hospital, Turku, Finland
3 Institute of Biomedicine, University of Turku, Turku, Finland
4 The UKK Institute for Health Promotion Research, Tampere, Finland
5 Department of Biostatistics, University of Turku and Turku University Hospital, Turku, Finland
6 Faculty of Medicine and Health Technology, Tampere University, Tampere, Finland

*Corresponding author: Jooa Norha, Turku PET Centre, University of Turku and Turku University Hospital, P.O. Box 52, 20521 Turku, Finland, E-mail: [jooa.norha@utu.fi](mailto:jooa.norha@utu.fi)

**Contents**

Supplemental Table S1. Intervention effects on the echocardiographic measures.

Supplemental Table S2. Differences in body mass index by sufficient-quality exercise global longitudinal strain data.

Supplemental Table S3. Results of the additional analysis.

Supplemental Figure S1. Residual histograms of the linear mixed models.

Supplemental Table S4. Correlation coefficients between the changes during the intervention.

Supplemental Table S5. Correlation coefficients between the changes (Δ) in global longitudinal strain (GLS) during exercise and the changes in sedentary behaviour and physical activities.

Supplemental Appendix S1. CONSORT Checklist.

Supplemental Table S1. Intervention effects on the resting echocardiographic variables. The estimates correspond to Figures 2 and 3.

|  | Intervention |  | Control |  |  |  |  |
| --- | --- | --- | --- | --- | --- | --- | --- |
|  | Pre | Post | Pre | Post | p-values | Group difference^a^ | Group x time p-value |
| Septum, mm | 8.5 (8.1, 8.9) | 8.4 (8.0, 8.8) | 8.7 (8.3, 9.1) | 8.6 (8.2, 9.0) | Group 0.428 Time 0.484 **Sex <0.001** | -0.1 (-0.7, 0.6) | 0.874 |
| Posterior wall, mm | 8.7 (8.3, 9.1) | 8.8 (8.4, 9.2) | 8.7 (8.3, 9.0) | 8.4 (8.0, 8.8) | Group 0.328 Time 0.614 **Sex <0.001** | 0.4 (-0.2, 1.0) | 0.236 |
| Relative wall thickness | 0.33 (0.31, 0.34) | 0.33 (0.32, 0.35) | 0.33 (0.32, 0.35) | 0.32 (0.31, 0.34) | Group 0.880 Time 0.859 Sex 0.072 | 0.02 (-0.01, 0.05) | 0.200 |
| LV end-diastolic diameter, mm | 53.2 (51.7, 54.8) | 52.6 (51.0, 54.2) | 52.0 (50.4, 53.5) | 52.2 (50.6, 53.8) | Group 0.446 Time 0.592 **Sex 0.008** | -0.9 (-2.4, 0.5) | 0.203 |
| LV mass, g | 199.5 (181.8, 217.3) | 189.8 (175.0, 204.7) | 188.6 (170.8, 206.5) | 183.0 (168.0, 198.1) | Group 0.417 Time 0.074 **Sex <0.001** | -4.1 (-21.0, 12.7) | 0.627 |
| LV mass index, g/m^2^ | 82.0 (76.4, 87.6) | 80.1 (75.3, 85.0) | 78.4 (72.7, 84.1) | 76.9 (72.0, 81.7) | Group 0.302 Time 0.309 **Sex 0.015** | -0.3 (-6.9, 6.3) | 0.920 |
| Left atrial diameter, mm | 39.8 (38.2, 41.5) | 40.5 (38.9, 42.2) | 40.3 (38.6, 41.9) | 40.1 (38.4, 41.8) | Group 0.983 Time 0.483 Sex 0.063 | 0.8 (-0.7, 2.3) | 0.270 |
| Left atrial end-systolic volume index, ml/m^2^ | 27.5 (24.8, 30.2) | 28.1 (25.3, 30.9) | 26.3 (23.5, 29) | 25.3 (22.3, 28.3) | Group 0.247 Time 0.844 Sex 0.549 | 1.6 (-2.5, 5.6) | 0.439 |
| Aortic root, mm | 33.6 (32.5, 34.7) | 33.5 (32.3, 34.6) | 34.2 (33.1, 35.3) | 33.8 (32.7, 35.0) | Group 0.523 Time 0.376 Sex <0.001 | 0.3 (-1.1, 1.6) | 0.705 |
| Early diastolic flow velocity (E), cm/s | 0.66 (0.60, 0.72) | 0.64 (0.58, 0.70) | 0.70 (0.64, 0.76) | 0.72 (0.66, 0.78) | Group 0.113 Time 0.962 Sex 0.127 | -0.04 (-0.12, 0.03) | 0.247 |
| Atrial contraction flow velocity (A), cm/s | 0.76 (0.71, 0.82) | 0.74 (0.68, 0.80) | 0.71 (0.66, 0.77) | 0.72 (0.66, 0.78) | Group 0.352 Time 0.614 Sex 0.168 | -0.03 (-0.09, 0.03) | 0.400 |
| E/A | 0.91 (0.81, 1.00) | 0.88 (0.79, 0.98) | 1.01 (0.91, 1.10) | 1.02 (0.92, 1.12) | Group 0.056 Time 0.869 Sex 0.953 | -0.03 (-0.15, 0.09) | 0.598 |
| Lateral E/e' | 7.6 (7, 8.3) | 8 (7.2, 8.8) | 7.4 (6.8, 8.1) | 7.4 (6.5, 8.2) | Group 0.382 Time 0.483 Sex 0.735 | 0.4 (-0.6, 1.5) | 0.399 |
| End-diastolic volume, ml | 91.2 (84.4, 98) | 87.2 (80.2, 94.2) | 92.5 (85.7, 99.3) | 89.3 (82, 96.6) | Group 0.690 Time 0.172 **Sex <0.001** | -0.8 (-11.2, 9.6) | 0.873 |
| End-systolic volume, ml | 36.5 (32.0, 41.0) | 33.4 (30.0, 36.8) | 35.3 (30.8, 39.8) | 36.1 (32.5, 39.7) | Group 0.737 Time 0.522 **Sex <0.001** | -3.9 (-11.1, 3.2) | 0.276 |
| Ejection fraction, % | 62.9 (61.2, 64.5) | 62.8 (61.2, 64.5) | 62.8 (61.2, 64.4) | 63.7 (62, 65.5) | Group 0.652 Time 0.520 Sex 0.203 | -1 (-3.9, 2) | 0.514 |
| Global longitudinal strain, % | -17.7 (-18.6, -16.8) | -18.3 (-19.1, -17.4) | -18.8 (-19.7, -17.9) | -18.8 (-19.7, -17.8) | Group 0.156 Time 0.380 **Sex 0.011** | -0.6 (-1.9, 0.7) | 0.391 |
| Stroke volume, ml | 54.7 (50.1, 59.3) | 54.1 (49.4, 58.8) | 57.2 (52.6, 61.8) | 53.5 (48.6, 58.5) | Group 0.731 Time 0.243 **Sex 0.006** | 3.1 (-4.1, 10.3) | 0.356 |
| Cardiac output, ml/min | 3807.8 (3478.8, 4136.8) | 3743.4 (3408.6, 4078.1) | 4096 (3764.5, 4427.5) | 3697.3 (3343.2, 4051.4) | Group 0.534 Time 0.110 **Sex 0.007** | 334.2 (-236.4, 904.8) | 0.246 |

LV=left ventricle, EDD=end diastolic diameter, EDV=end diastolic volume, ESV=end systolic volume, LA=left atrium, EF=ejection fraction, GLS=global longitudinal strain, e’=lateral mitral annular velocity (cm/s). Statistically significant p-values are bolded. ^a^=difference in change between the groups (the change in the control group subtracted from the change in the intervention group)

Supplemental Table S2. Mean body mass index (95% CI) in participants with and without sufficient-quality global longitudinal strain (GLS) measurements during exercise testing at different stages of the exercise test at baseline.

|  | Has sufficient-quality GLS data | n | Does not have sufficient-quality GLS data | n | p-value* |
| --- | --- | --- | --- | --- | --- |
| At 25 W | 30.6 (29.4, 31.8) | 44 | 33.9 (32.1, 35.6) | 20 | 0.004 |
| At 50 W | 30.2 (29.0, 31.4) | 42 | 34.3 (32.7, 35.9) | 22 | 0.0001 |
| At 75 W | 30.1 (28.9, 31.4) | 38 | 33.8 (32.3, 35.3) | 26 | 0.0004 |
| At 100 W | 30.5 (29.0, 32.0) | 32 | 32.7 (31.3, 34.2) | 32 | 0.033 |
| At 125 W | 30.7 (29.0, 32.4) | 25 | 32.2 (30.9, 33.6) | 39 | 0.16 |

*One-way ANOVA for group difference in body mass index.

Supplemental Table S3. Results of the additional analysis. Less sedentary group represents the participants who successfully reduced sedentary behaviour by at least 3%-points and continuously sedentary represent the participants who either increased their sedentary time or decreased it by less than 3%-points.

|  | Less sedentary |  | Continuously sedentary |  |  |  |  |
| --- | --- | --- | --- | --- | --- | --- | --- |
|  | Pre | Post | Pre | Post | p-values | Group difference^a^ | Group x time p-value |
| Septum, mm | 8.6 (8.2, 9) | 8.5 (8.1, 8.9) | 8.6 (8.2, 9) | 8.5 (8, 8.9) | Group 0.914 Time 0.469 **Sex <0.001** | 0 (-0.7, 0.7) | 0.947 |
| Posterior wall, mm | 8.8 (8.4, 9.1) | 8.7 (8.3, 9.1) | 8.6 (8.2, 9) | 8.5 (8.1, 8.9) | Group 0.345 Time 0.650 **Sex <0.001** | 0 (-0.6, 0.6) | 0.959 |
| Relative wall thickness | 0.33 (0.31, 0.34) | 0.33 (0.32, 0.35) | 0.33 (0.32, 0.35) | 0.32 (0.31, 0.34) | Group 0.909 Time 0.837 Sex 0.078 | 0.02 (-0.01, 0.04) | 0.275 |
| LV end-diastolic diameter, mm | 53.5 (52, 55) | 52.5 (51, 54.1) | 51.6 (50, 53.2) | 52.3 (50.7, 53.9) | Group 0.314 Time 0.677 **Sex 0.008** | -1.7 (-3.1, -0.3) | **0.020** |
| LV mass, g | 202.7 (185.4, 219.9) | 188.2 (173.6, 202.7) | 184.8 (166.8, 202.8) | 185 (169.6, 200.4) | Group 0.333 Time 0.088 **Sex <0.001** | -14.7 (-31.2, 1.7) | 0.078 |
| LV mass index, g/m^2^ | 83.4 (78, 88.9) | 78.6 (73.8, 83.5) | 76.7 (71, 82.3) | 78.2 (73.1, 83.3) | Group 0.285 Time 0.298 **Sex 0.017** | -6.4 (-12.6, -0.1) | **0.045** |
| Left atrial diameter, mm | 39.8 (38.2, 41.4) | 40.4 (38.8, 42) | 40.3 (38.6, 42) | 40.3 (38.6, 42) | Group 0.875 Time 0.471 Sex 0.064 | 0.5 (-1, 2.1) | 0.468 |
| Left atrial end-systolic volume index, ml/m^2^ | 27.1 (24.3, 29.8) | 27.3 (24.4, 30.1) | 26.7 (23.9, 29.5) | 26.2 (23.3, 29.2) | Group 0.693 Time 0.893 Sex 0.629 | 0.7 (-3.3, 4.7) | 0.741 |
| Aortic root, mm | 33.4 (32.3, 34.4) | 33.4 (32.3, 34.5) | 34.5 (33.4, 35.7) | 33.9 (32.8, 35.1) | Group 0.228 Time 0.349 **Sex <0.001** | 0.6 (-0.7, 1.9) | 0.357 |
| Early diastolic flow velocity (E), cm/s | 0.69 (0.63, 0.74) | 0.65 (0.59, 0.71) | 0.67 (0.61, 0.74) | 0.71 (0.65, 0.77) | Group 0.544 Time 0.981 Sex 0.176 | -0.07 (-0.14, 0.00) | 0.056 |
| Atrial contraction flow velocity (A), cm/s | 0.76 (0.71, 0.82) | 0.73 (0.68, 0.78) | 0.71 (0.66, 0.77) | 0.73 (0.67, 0.79) | Group 0.468 Time 0.681 Sex 0.159 | -0.05 (-0.12, 0.01) | 0.092 |
| E/A | 0.93 (0.84, 1.03) | 0.91 (0.82, 1.01) | 0.98 (0.89, 1.08) | 0.99 (0.89, 1.09) | Group 0.304 Time 0.859 Sex 0.931 | -0.02 (-0.14, 0.09) | 0.678 |
| Lateral E/e' | 7.5 (6.9, 8.1) | 7.9 (7.1, 8.7) | 7.5 (6.9, 8.2) | 7.6 (6.7, 8.4) | Group 0.787 Time 0.470 Sex 0.789 | 0.3 (-0.7, 1.4) | 0.526 |
| End-diastolic volume, ml | 91.0 (84.4, 97.7) | 85.4 (78.5, 92.3) | 92.7 (85.8, 99.6) | 91.4 (84.1, 98.7) | Group 0.365 Time 0.185 **Sex <0.001** | -4.3 (-14.7, 6.1) | 0.411 |
| End-systolic volume, ml | 36.7 (32.3, 41.1) | 34.4 (31, 37.8) | 35.1 (30.5, 39.7) | 35.0 (31.4, 38.6) | Group 0.822 Time 0.508 **Sex <0.001** | -2.2 (-9.5, 5) | 0.539 |
| Ejection fraction, % | 62.4 (60.8, 64) | 62.8 (61.2, 64.4) | 63.2 (61.6, 64.9) | 63.8 (62, 65.5) | Group 0.329 Time 0.543 Sex 0.178 | -0.1 (-3.1, 2.9) | 0.940 |
| Global longitudinal strain, % | -18.3 (-19.2, -17.4) | -18.1 (-19, -17.2) | -18.1 (-19, -17.2) | -18.9 (-19.9, -18) | Group 0.559 Time 0.320 **Sex 0.014** | 1 (-0.3, 2.3) | 0.129 |
| Stroke volume, ml | 54.3 (49.9, 58.8) | 51.3 (46.6, 55.9) | 57.6 (53, 62.2) | 56.8 (51.9, 61.7) | Group 0.114 Time 0.293 **Sex 0.005** | -2.3 (-9.5, 5) | 0.532 |
| Cardiac output, ml/min | 3772.6 (3453.2, 4091.9) | 3600.6 (3271.3, 3930) | 4143.7 (3809.8, 4477.5) | 3870 (3518.6, 4221.5) | Group 0.094 Time 0.129 **Sex 0.006** | 101.7 (-476.9, 680.3) | 0.726 |

LV=left ventricle, EDD=end diastolic diameter, EDV=end diastolic volume, ESV=end systolic volume, LA=left atrium, EF=ejection fraction, GLS=global longitudinal strain, e’=lateral mitral annular velocity (cm/s). Statistically significant p-values are bolded. ^a^=difference in change between the groups (the change in the continuously sedentary group subtracted from the change in the less sedentary group).

Supplemental Figure S1. Studentised residuals of the statistically significant linear mixed models. A) Left ventricular (LV) mass index, B) Left ventricular end-diastolic diameter, Global longitudinal strain (GLS) at C) 25 W, D) 100 W, and C) 125 W during the exercise test. All of these residuals are from the additional analyses with the continuously sedentary vs. less sedentary groups.


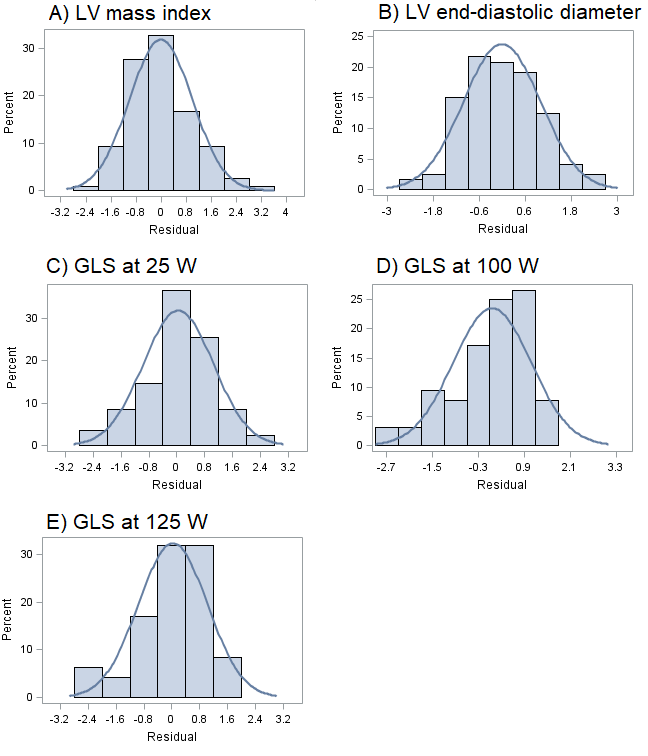


Supplemental Table S4. Pearson correlations between the changes (Δ) in echocardiographic variables and the changes in sedentary behaviour and physical activities.

|  | Δ Septum | Δ Post wall | Δ RWT | Δ LV mass | Δ LV mass index | Δ LV EDD | Δ LV EF | Δ LV EDV | Δ LV ESV | Δ Stroke volume | Δ GLS | Δ E | Δ A | Δ E/A | Δ E/e' | Δ LA diameter | Δ LAESV index | Δ Aortic root | Δ SB | Δ Standing | Δ LPA | Δ MVPA | Δ Total PA | Δ Steps | Δ Breaks | Δ Body fat % |
| --- | --- | --- | --- | --- | --- | --- | --- | --- | --- | --- | --- | --- | --- | --- | --- | --- | --- | --- | --- | --- | --- | --- | --- | --- | --- | --- |
| Δ Post wall | 0.12 | -- |  |  |  |  |  |  |  |  |  |  |  |  |  |  |  |  |  |  |  |  |  |  |  |  |
| Δ RWT | 0.13 | 0.95** | -- |  |  |  |  |  |  |  |  |  |  |  |  |  |  |  |  |  |  |  |  |  |  |  |
| Δ LV mass | .70^**^ | .41^**^ | 0.24 | -- |  |  |  |  |  |  |  |  |  |  |  |  |  |  |  |  |  |  |  |  |  |  |
| Δ LV mass index | .68^**^ | .47^**^ | 0.29* | .93^**^ | -- |  |  |  |  |  |  |  |  |  |  |  |  |  |  |  |  |  |  |  |  |  |
| Δ LV EDD | -0.05 | -.29^*^ | -0.56** | .41^**^ | .36^**^ | -- |  |  |  |  |  |  |  |  |  |  |  |  |  |  |  |  |  |  |  |  |
| Δ LV EF | 0.00 | -0.12 | -0.24 | 0.14 | 0.14 | .37^**^ | -- |  |  |  |  |  |  |  |  |  |  |  |  |  |  |  |  |  |  |  |
| Δ LV EDV | 0.17 | 0.15 | 0.09 | .33^*^ | .34^*^ | 0.15 | 0.04 | -- |  |  |  |  |  |  |  |  |  |  |  |  |  |  |  |  |  |  |
| Δ LV ESV | 0.25 | 0.01 | -0.01 | .33^*^ | 0.26 | 0.14 | -0.17 | .71^**^ | -- |  |  |  |  |  |  |  |  |  |  |  |  |  |  |  |  |  |
| Δ Stroke volume | -0.02 | 0.20 | 0.14 | 0.12 | 0.22 | 0.07 | 0.24 | .70^**^ | -0.01 | -- |  |  |  |  |  |  |  |  |  |  |  |  |  |  |  |  |
| Δ GLS | 0.14 | -0.01 | 0.05 | 0.11 | 0.06 | -0.07 | -0.13 | -0.05 | 0.10 | -0.13 | -- |  |  |  |  |  |  |  |  |  |  |  |  |  |  |  |
| Δ E | -0.02 | -0.08 | -0.12 | 0.06 | 0.15 | 0.16 | 0.15 | .32^*^ | 0.11 | .34^*^ | -.29^*^ | -- |  |  |  |  |  |  |  |  |  |  |  |  |  |  |
| Δ A | -0.08 | 0.01 | -0.01 | 0.02 | 0.05 | 0.06 | -0.11 | 0.11 | -0.03 | 0.19 | -0.13 | .45^**^ | -- |  |  |  |  |  |  |  |  |  |  |  |  |  |
| Δ E/A | 0.06 | -0.05 | -0.09 | 0.09 | 0.11 | 0.14 | 0.23 | 0.21 | 0.17 | 0.12 | -0.19 | .51^**^ | -.49^**^ | -- |  |  |  |  |  |  |  |  |  |  |  |  |
| Δ E/e' | 0.17 | 0.18 | 0.16 | 0.18 | 0.17 | 0.02 | -0.01 | .35^**^ | 0.18 | .34^*^ | -0.07 | .35^**^ | 0.10 | 0.23 | -- |  |  |  |  |  |  |  |  |  |  |  |
| Δ LA diameter | -0.04 | -0.12 | -0.18 | 0.13 | 0.12 | 0.21 | 0.02 | 0.15 | 0.15 | 0.06 | 0.01 | .28^*^ | 0.03 | 0.26 | 0.10 | -- |  |  |  |  |  |  |  |  |  |  |
| Δ LAESV index | -0.16 | 0.05 | 0.08 | -0.16 | -0.15 | -0.18 | 0.17 | 0.07 | -0.09 | 0.20 | -0.16 | .30^*^ | -0.17 | .43^**^ | 0.16 | 0.10 | -- |  |  |  |  |  |  |  |  |  |
| Δ Aortic root | 0.08 | -0.09 | -0.08 | -0.01 | -0.05 | -0.05 | -0.28* | -0.27* | -0.17 | -0.22 | 0.01 | -0.02 | 0.07 | -0.14 | -0.21 | 0.04 | 0.02 | -- |  |  |  |  |  |  |  |  |
| Δ SB | -0.04 | -0.01 | -0.08 | 0.12 | 0.13 | 0.16 | 0.01 | 0.05 | 0.03 | 0.03 | -0.15 | 0.09 | 0.25 | -0.12 | -0.22 | -0.16 | -0.04 | -0.17 | -- |  |  |  |  |  |  |  |
| Δ Standing | 0.16 | -0.01 | 0.08 | -0.08 | -0.07 | -0.19 | -0.06 | 0.05 | -0.05 | 0.09 | 0.14 | -0.02 | -0.22 | 0.16 | .28^*^ | 0.02 | 0.00 | -0.11 | -.79^**^ | -- |  |  |  |  |  |  |
| Δ LPA | -0.23 | 0.05 | 0.12 | -0.26 | -.32^*^ | -0.22 | 0.05 | -0.05 | 0.02 | -0.07 | 0.13 | -0.08 | -0.14 | 0.02 | 0.05 | 0.18 | 0.26 | 0.38** | -.69^**^ | 0.24 | -- |  |  |  |  |  |
| Δ MVPA | 0.08 | -0.02 | -0.04 | 0.07 | 0.08 | 0.08 | 0.03 | -0.16 | -0.02 | -0.17 | 0.04 | -0.15 | -0.18 | 0.03 | 0.08 | 0.24 | -0.15 | 0.28* | -.69^**^ | 0.25 | .46^**^ | -- |  |  |  |  |
| Δ Total PA | -0.08 | 0.02 | 0.05 | -0.10 | -0.14 | -0.09 | 0.04 | -0.13 | 0.01 | -0.15 | 0.10 | -0.14 | -0.18 | 0.02 | 0.06 | 0.23 | 0.07 | 0.38** | -.81^**^ | .29^*^ | .86^**^ | .85^**^ | -- |  |  |  |
| Δ Steps | -0.05 | -0.13 | -0.11 | -0.07 | -0.08 | 0.03 | -0.08 | -0.11 | -0.03 | -0.10 | 0.17 | -0.15 | -0.07 | -0.07 | 0.01 | 0.18 | -0.22 | 0.17 | -.55^**^ | 0.20 | .37^**^ | .79^**^ | .67^**^ | -- |  |  |
| Δ Breaks | -0.06 | -0.16 | -0.14 | -0.19 | -0.19 | -0.02 | -0.02 | -0.03 | 0.13 | -0.13 | 0.00 | -0.02 | 0.03 | -0.04 | -0.13 | 0.02 | -0.17 | 0.19 | -.38^**^ | 0.17 | .35^**^ | .39^**^ | .45^**^ | .48^**^ | -- |  |
| Δ Body fat % | -0.01 | 0.33* | 0.31* | 0.16 | 0.09 | -0.06 | 0.14 | -0.06 | -0.08 | 0.00 | 0.15 | -0.20 | -0.21 | 0.04 | -0.13 | 0.01 | 0.01 | 0.10 | 0.12 | -0.21 | 0.09 | -0.07 | 0.02 | -0.12 | -0.22 | -- |
| Δ Body weight | 0.39** | 0.13 | 0.12 | 0.29* | 0.28* | -0.07 | 0.07 | 0.11 | 0.06 | 0.09 | -0.07 | 0.00 | -0.19 | 0.20 | 0.06 | 0.03 | -0.12 | -0.05 | 0.26 | -0.1 | -0.37** | -0.17 | -0.31* | -0.21 | -0.17 | 0.22 |

Septum = septal wall thickness. Post wall = left ventricular posterior wall thickness. LV = left ventricle. EDD = end-diastolic diameter. EF = ejection fraction. EDV = end-diastolic volume. ESV = end-systolic volume. GLS = left ventricular global longitudinal strain. E = peak early diastolic transmitral flow velocity. A = transmitral flow velocity during atrial contraction. LA = left atrium. LAESV = left atrial end-systolic volume index. SB = sedentary behaviour. LPA = light physical activity. MVPA = moderate-to-vigorous physical activity. Total PA = total physical activity. *p<0.05, **p<0.01

Supplemental Table S5. Pearson correlations between the changes (Δ) in global longitudinal strain (GLS) during exercise and the changes in sedentary behaviour and physical activities.

|  | Δ GLS at 25 W | Δ GLS at 50 W | Δ GLS at 75 W | Δ GLS at 100 W | Δ GLS at 125 W |
| --- | --- | --- | --- | --- | --- |
| Δ SB | 0.26 | 0.25 | 0.03 | 0.37 | 0.44 |
| Δ Standing | -0.25 | -0.17 | 0.25 | -0.56** | -0.54* |
| Δ LPA | -0.02 | -0.03 | -0.39* | -0.03 | -0.02 |
| Δ MVPA | -0.24 | -0.32 | -0.15 | -0.15 | -0.24 |
| Δ Total PA | -0.15 | -0.21 | -0.29 | -0.07 | -0.15 |
| Δ Steps | -0.41* | -0.46* | -0.15 | -0.05 | -0.22 |
| Δ Breaks | -0.04 | -0.25 | -0.07 | 0.05 | -0.18 |
| Δ Body fat % | 0.27 | 0.29 | -0.01 | -0.12 | -0.02 |
| Δ Body weight | -0.12 | -0.04 | -0.21 | -0.13 | -0.12 |

SB = sedentary behaviour. LPA = light physical activity. MVPA = moderate-to-vigorous physical activity. Total PA = total physical activity. *p<0.05, **p<0.01

Supplemental Appendix S1. CONSORT Checklist.


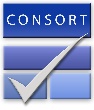
CONSORT 2010 checklist of information to include when reporting a randomised trial*

| Section/Topic | Item No | Checklist item | Reported on page No |
| --- | --- | --- | --- |
| Title and abstract | | | |
|  | 1a | Identification as a randomised trial in the title | 1 |
|  | 1b | Structured summary of trial design, methods, results, and conclusions (for specific guidance see CONSORT for abstracts) | 2 |
| Introduction | | | |
| Background and objectives | 2a | Scientific background and explanation of rationale | 3 |
|  | 2b | Specific objectives or hypotheses | 3 |
| Methods | | | |
| Trial design | 3a | Description of trial design (such as parallel, factorial) including allocation ratio | 4, 5 |
|  | 3b | Important changes to methods after trial commencement (such as eligibility criteria), with reasons | - |
| Participants | 4a | Eligibility criteria for participants | 4 |
|  | 4b | Settings and locations where the data were collected | 4 |
| Interventions | 5 | The interventions for each group with sufficient details to allow replication, including how and when they were actually administered | 5 |
| Outcomes | 6a | Completely defined pre-specified primary and secondary outcome measures, including how and when they were assessed | 6-7 |
|  | 6b | Any changes to trial outcomes after the trial commenced, with reasons | - |
| Sample size | 7a | How sample size was determined | 7 |
|  | 7b | When applicable, explanation of any interim analyses and stopping guidelines | - |
| Randomisation: |  |  | 5 |
| Sequence generation | 8a | Method used to generate the random allocation sequence |  |
|  | 8b | Type of randomisation; details of any restriction (such as blocking and block size) | 5 |
| Allocation concealment mechanism | 9 | Mechanism used to implement the random allocation sequence (such as sequentially numbered containers), describing any steps taken to conceal the sequence until interventions were assigned | 5 |
| Implementation | 10 | Who generated the random allocation sequence, who enrolled participants, and who assigned participants to interventions | 5 |
| Blinding | 11a | If done, who was blinded after assignment to interventions (for example, participants, care providers, those assessing outcomes) and how | - |
|  | 11b | If relevant, description of the similarity of interventions | 5 |
| Statistical methods | 12a | Statistical methods used to compare groups for primary and secondary outcomes | 7-8 |
|  | 12b | Methods for additional analyses, such as subgroup analyses and adjusted analyses | 7-8 |
| Results | | | |
| Participant flow (a diagram is strongly recommended) | 13a | For each group, the numbers of participants who were randomly assigned, received intended treatment, and were analysed for the primary outcome | Fig 1 |
|  | 13b | For each group, losses and exclusions after randomisation, together with reasons | Fig 1 |
| Recruitment | 14a | Dates defining the periods of recruitment and follow-up | 4 |
|  | 14b | Why the trial ended or was stopped | - |
| Baseline data | 15 | A table showing baseline demographic and clinical characteristics for each group | Tables 1, 2 |
| Numbers analysed | 16 | For each group, number of participants (denominator) included in each analysis and whether the analysis was by original assigned groups | Fig 1 |
| Outcomes and estimation | 17a | For each primary and secondary outcome, results for each group, and the estimated effect size and its precision (such as 95% confidence interval) | Figures 2-4, Table 3 |
|  | 17b | For binary outcomes, presentation of both absolute and relative effect sizes is recommended | - |
| Ancillary analyses | 18 | Results of any other analyses performed, including subgroup analyses and adjusted analyses, distinguishing pre-specified from exploratory | Fig 4, Table 3 |
| Harms | 19 | All important harms or unintended effects in each group (for specific guidance see CONSORT for harms) | - |
| Discussion | | | |
| Limitations | 20 | Trial limitations, addressing sources of potential bias, imprecision, and, if relevant, multiplicity of analyses | 10-15 |
| Generalisability | 21 | Generalisability (external validity, applicability) of the trial findings | 10-15 |
| Interpretation | 22 | Interpretation consistent with results, balancing benefits and harms, and considering other relevant evidence | 10-15 |
| Other information | | |  |
| Registration | 23 | Registration number and name of trial registry | 4 |
| Protocol | 24 | Where the full trial protocol can be accessed, if available | 4 |
| Funding | 25 | Sources of funding and other support (such as supply of drugs), role of funders | 15 |

Citation: Schulz KF, Altman DG, Moher D, for the CONSORT Group. CONSORT 2010 Statement: updated guidelines for reporting parallel group randomised trials. BMC Medicine. 2010;8:18.
© 2010 Schulz et al. This is an Open Access article distributed under the terms of the Creative Commons Attribution License (<http://creativecommons.org/licenses/by/2.0>), which permits unrestricted use, distribution, and reproduction in any medium, provided the original work is properly cited.

*We strongly recommend reading this statement in conjunction with the CONSORT 2010 Explanation and Elaboration for important clarifications on all the items. If relevant, we also recommend reading CONSORT extensions for cluster randomised trials, non-inferiority and equivalence trials, non-pharmacological treatments, herbal interventions, and pragmatic trials. Additional extensions are forthcoming: for those and for up-to-date references relevant to this checklist, see [www.consort-statement.org](http://www.consort-statement.org).
